# Molecular epidemiology and risk factors of *Stenotrophomonas maltophilia* infections in a Chinese teaching hospital

**DOI:** 10.1186/s12866-020-01985-3

**Published:** 2020-09-29

**Authors:** Zhongliang Duan, Juanxiu Qin, Yao Liu, Cui Li, Chunmei Ying

**Affiliations:** 1 Department of Clinical Laboratory, Obstetrics and Gynecology Hospital of Fudan University, 419 Fangxie Road, Shanghai, 200011 China; 2 Department of Laboratory Medicine, Renji Hospital, School of Medicine, Shanghai Jiao Tong University, Shanghai, 200001 China

**Keywords:** *Stenotrophomonas maltophilia*, MLST, Biofilm, Epidemiology

## Abstract

**Background:**

*Stenotrophomonas maltophilia* (*S. maltophilia*) is an important opportunistic pathogen that can be isolated in hospitals. With the abuse of broad spectrum antibiotics and invasive surgical devices, the rate of *S. maltophilia* infection is increasing every year. This study was an epidemiological analysis of the clinical and molecular characteristics of *S. maltophilia* infection in a Chinese teaching hospital. The goal was to obtain a comprehensive understanding of the status of *S. maltophilia* infection to provide strong epidemiological data for the prevention and treatment of *S. maltophilia* infection.

**Results:**

A total of 93 isolates from Renji Hospital affiliated with the Shanghai Jiaotong University School of Medicine were included, in which 62 isolates were from male patients. In addition, 81 isolates were isolated from sputum samples. A total of 86 patients had underlying diseases. All patients received antibiotics. Multilocus sequence typing (MLST) analysis indicated that 61 different sequence types (STs) were found (including 45 novel STs), and MLST did not show significantly dominant STs. Pulsed field gel electrophoresis (PFGE) results showed that 93 isolates could be divided into 73 clusters, and they also showed weak genetic linkages between isolates. The resistant rates to trimethoprim/sulfamethoxazole (TMP/SMX) and levofloxacin were 9.7 and 4.3%, respectively, and all isolates were susceptible to minocycline. Four virulence gene’s loci *Stmpr1*, *Stmpr2*, *Smf-1*, and *Smlt3773* were positive in 79.6, 91.4, 94.6, and 52.7% of the isolates, respectively. Three biofilm genes *rmlA*, *spgM*, and *rpfF* were positive in 82.8, 92.5, and 64.5% of the isolates, respectively. Mean biofilm forming level of OD_492_ was 0.54 ± 0.49. We did not find any significant difference between different genders and different age-groups. We retrospectively analyzed data from patients in the intensive care unit (ICU) and the control group. The independent risk factors of those who were infected in the ICU included immunosuppression and the increased antibiotic usage.

**Conclusions:**

Most of the patients had prior medical usage histories and baseline diseases. The positive rate of virulence genes was high, the drug resistance rate of *S. maltophilia* was low, and the biofilm formation ability was strong. The increased use of antibiotics was an independent risk factor for *S. maltophilia* infection, which should receive more attention. No obvious clonal transmissions were found in the same departments.

## Background

*S. maltophilia*, called *Xanthomonas maltophilia* previously, is a nonfermentative, gram-negative aerobic bacilli. It is a cosmopolitan bacteria and originally was a plant pathogen, being ubiquitous in natural environments like water, soils, and plants [1]. *S. maltophilia* also can be found in medical settings, including numerous hospital devices, such as dialysis devices, blood pressure monitors, faucets, sphygmomanometers, disinfectants, and ventilators. It has the ability to transmit between patients or from patients to healthy people [2]. Clinical evidence has shown that *S. maltophilia* can cause nosocomial infections in immunocompromised hosts, such as respiratory system infections, joint infections, and skin infections [3]. Wu et al. [4] also found that the dominant flora in keratitis infection was *S. maltophilia.* In several of the China Antimicrobial Surveillance Network (CHINET) reports, among the nonfermentative gram-negative bacilli, *S. maltophilia* was the third largest per year, just after *Pseudomonas aeruginosa* and *Acinetobacter baumannii*, and the number of clinical isolates showed an upward trend [5].

With the abuse of different types of antibiotics, chemotherapy drugs, and immunosuppressants, and the widespread use of invasive exploration equipment, the isolation and infection rates of *S. maltophilia* in hospitals have continued to increase. Brooke [2] reported that particular attention had to be given to inpatients receiving immunosuppression. *S. maltophilia* is intrinsically resistant to several kinds of antibiotics because of its various resistance mechanisms. It can produce a penicillinase (L1) and a cephalosporinase (L2), which makes it easily resistant to β-lactam antibiotics, specifically carbapenems [6]. It can produce aminoglycoside modifying enzymes, which makes it resistant to aminoglycoside drugs to a certain degree, and the efflux pump system also makes it resistant to a variety of antimicrobial agents [7]. Hence, the continuous emergence of multidrug-resistant isolates of *S. maltophilia* has brought significant challenges for the treatment of serious *S. maltophilia* infection [5].

Few studies have investigated the comprehensive clinical and molecular characteristics of *S. maltophilia* in Shanghai. Therefore, in this study, we used multilocus sequence typing (MLST) and pulsed field gel electrophoresis (PFGE) to analyze the molecular epidemiological characteristics of *S. maltophilia* isolated from the Renji Hospital affiliated with the Shanghai Jiaotong University School of Medicine. We also analyzed the risk factors of *S. maltophilia* infection from patients in an intensive care unit (ICU). We collected clinical information of the related patients, and at the same time, detected the virulence genes and biofilm genes of *S. maltophilia*. The aim of this study was to develop an intuitive description of the epidemic situation of the strain in clinic. This research provided the necessary groundwork for basic and mechanical studies and provided support for the prevention and treatment of *S. maltophilia* infection.

## Results

### Patients and bacteria isolates

**Fig. 1:**
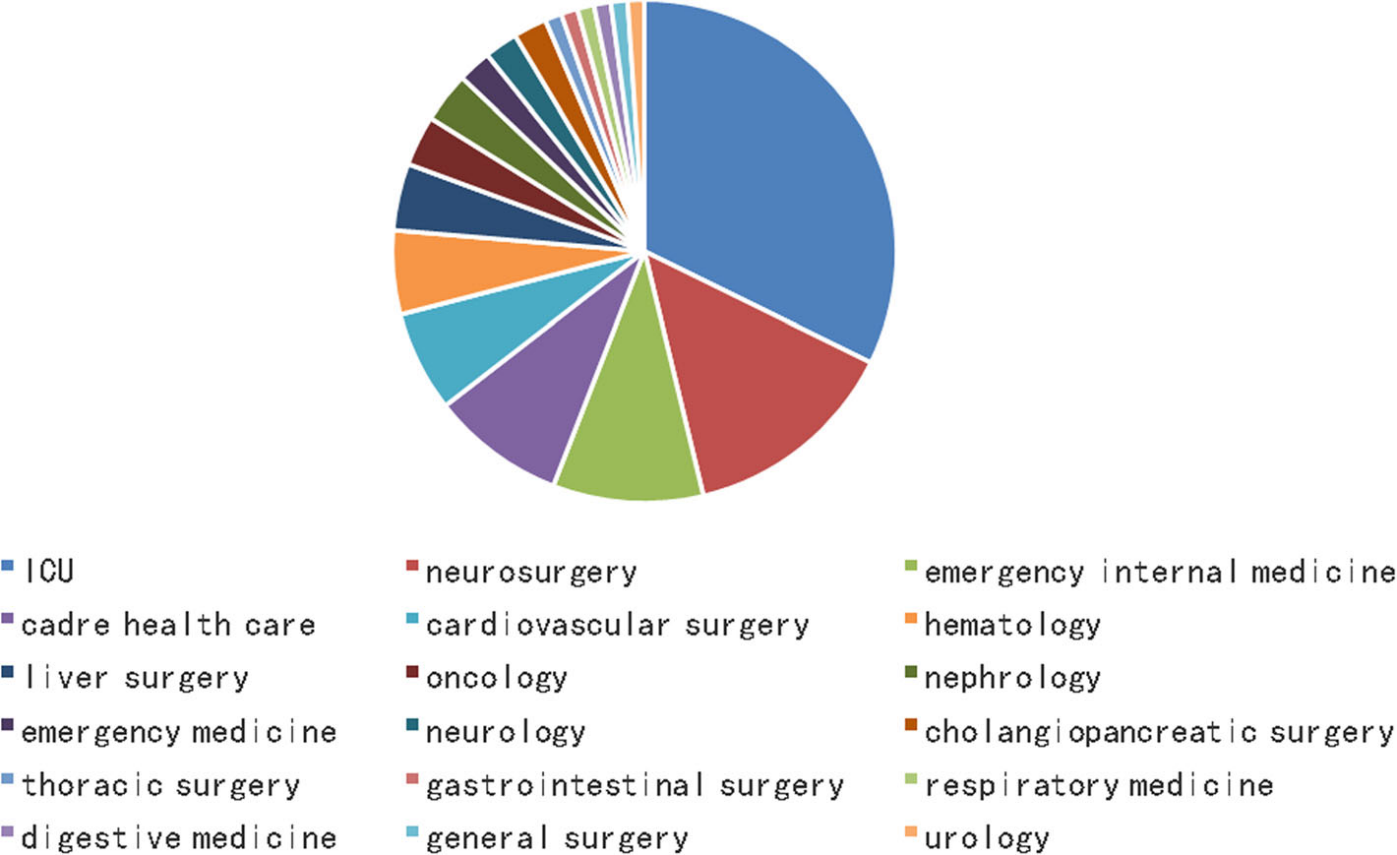
The distribution of *S. maltophilia* in the different departments. The pie graph shows the distribution of the wards, among the top three wards. Blue, red, and yellow represent the ICU, neurosurgery, and emergency internal medicine ward, respectively

We collected patients’ clinical information and isolated a total of 93 isolates of nonrepetitive *S. maltophilia*. Among them, we isolated 30 isolates of *S. maltophilia* from the ICU, and the rest were from a range of hospital departments (Fig. 1), including 13 isolates from neurosurgery, 9 isolates from emergency internal medicine, 8 isolates from cadre health care, 6 isolates from cardiovascular surgery, 5 isolates from hematology, 4 isolates from liver surgery, 3 isolates from oncology, 3 isolates from nephrology, 2 isolates from emergency medicine, 2 isolates from neurology, 2 isolates from cholangio-pancreatic surgery, 1 from thoracic surgery, 1 from gastrointestinal surgery, 1 from respiratory medicine, 1 from digestive medicine, 1 from general surgery, and 1 from urology.

Among the patients with *S. maltophilia* infection, 62 males (66.7%) and 31 females (33.3%) were included. A total of 73 patients were age 60 years or older (78.5%). Before bacterial isolation, 35 patients (37.6%) were subjected to invasive examinations or treatments. Among all the *S. maltophilia* isolates, 81 isolates (87.1%) were isolated from sputum, 7 isolates (7.5%) from drains, 2 isolates from pleural effusion, 1 strain from ascites, 1 strain from urine, and 1 strain from blood.

Of the 93 patients, 7 (7.5%) had no basic diseases but did have fractures and malnutrition, and 86 (92.5%) had one to five underlying diseases. Of these, 25 had malignant tumors, 24 had hypertension, 12 had coronary heart disease, 10 had renal insufficiency, 5 had leukemia, 14 had head trauma, 20 had chronic bronchitis or pneumonia, and 12 had liver injury. All of the patients had a history of antibiotic use (one to six types), with an average of three antibiotics per person before isolation of *S. maltophilia*, of which 69 had used three or more antibiotics. The antibiotics primarily included cephalosporins (65/93), carbapenem (53/93), β-lactamase inhibitors (51/93), quinolones (35/93), glycopeptide (22/93), aminoglycosides (13/93), and tetracyclines (9/93) (Table 1).

**Table 1:** The clinical characteristics of the adult and pediatric patients

	Adults(***n*** = 93)
**Demographics**	
Age (year, average, range)	66.3 (16–99)
Gender: male	62 (66.7%)
**Baseline diseases, n (%)**	
Hypertension	24 (25.8)
Heart disease	12 (12.9)
Malignancy	25 (26.9)
Pulmonary disease	20 (21.5)
Liver disease	12 (12.9)
Leukemia	5 (5.4)
Head trauma	14 (15.1)
**Strain isolation n (%)**	
ICU	30 (32.3)
Sputum	81 (87.1)
**Invasive operation n (%)**	35 (37.6)
**Previous antibiotics usage n (%)**	
The number of antibiotics ≥3	69 (74.2)
Cephalosporins	65 (69.9)
Carbapenems	53 (57.0)
β-lactamase Inhibitors	51 (54.8)
Quinolones	35 (37.6)
Glycopeptides	22 (23.7)
Aminoglycosides	13 (14.0)

### MLST analysis

**Fig. 2:**
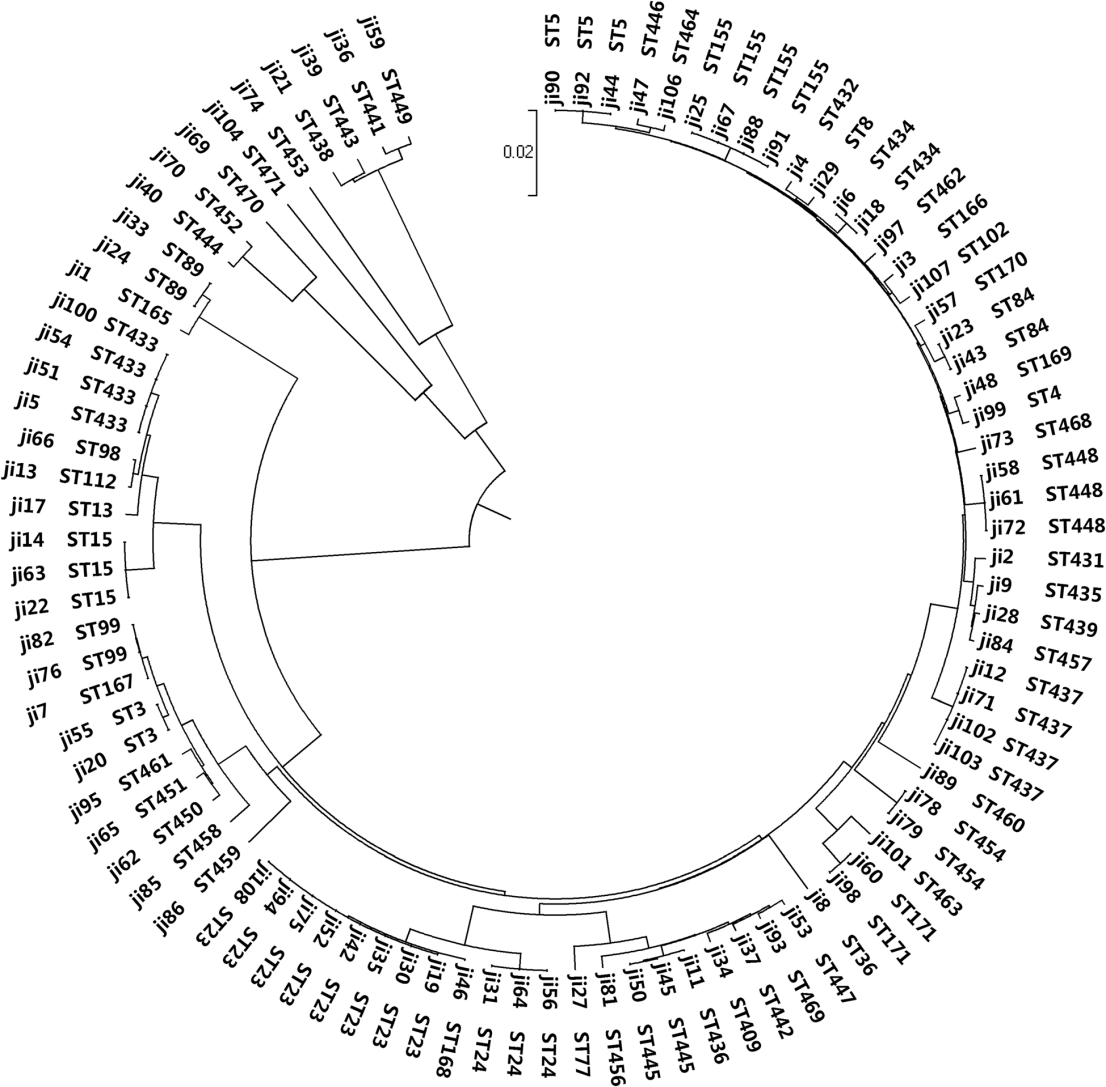
The MLST results of 93 *S. maltophilia* isolates. This is a neighbor-joining tree analysis for the concatenated data for all seven loci of the 93 isolates. The tree was rooted with the corresponding concatenate

The distribution of the clonal typing of *S. maltophilia* was relatively scattered. According to the different alleles, we assigned the isolates to 61 sequence types. Among them, 45 types of the 60 isolates were different from those published on the PubMLST database (recorded as STnew1-STnew45). The other 33 isolates consisted of existing types in the database, of which a relatively larger number was ST23 (*n* = 8). Some were isolates of ST5 (*n* = 3), ST15 (*n* = 3), ST24 (*n* = 3), ST3 (*n* = 2), ST84 (*n* = 2), ST89 (*n* = 2), and ST99 (*n* = 2), and some isolates were assigned to ST4, ST8, ST13, ST36, ST77, ST98, ST102, and ST112. The eight *S. maltophilia* isolates of ST23 were distributed in five different departments, and we classified the 30 isolates isolated from the ICU into 24 STs. We did not collected *S. maltophilia* isolates of the exact same sequence types in the other departments, which indicated the lack of obvious clonal transmission of *S. maltophilia* infections in this study [1]. The detailed results are shown in Fig. 2.

### PFGE typing results

According to the fragment diagnostic criteria of PFGE, PFGE types can be classified into a group or cluster if there are no more than three bands [8, 9]. In this study, the 93 SMA strains were scattered and could be divided into 73 clusters in this study. Among them, we divided 13 strains into the same cluster (from eight departments, not from the same department). We divided another five and two strains into the same cluster, but the others were all quite different. These results suggested that these isolates were not part of an outbreak in the department, and the detailed results are shown in Supplementary Figure 1.

### Virulence gene detection

The results of the virulence gene detection showed that the positive rates of the four virulence genes were 79.6% (74/93) for *Stmpr1*, 91.4% (85/93) for *Stmpr2*, 94.6% (88/93) for *Smf-1*, and 52.7% (49/93) for *Smlt3773*. There were 31 isolates of *S. maltophilia* that carried all four of the genes.

### Analysis of drug resistance

The resistance rates of *S. maltophilia* to levofloxacin and TMP/SMX were 4.3 and 9.7%, respectively. All of *S. maltophilia* isolates were susceptible to minocycline. Among these isolates, one isolate, numbered ji82, was resistant to both TMP/SMX and levofloxacin.

### Biofilm formation ability

**Fig. 3:**
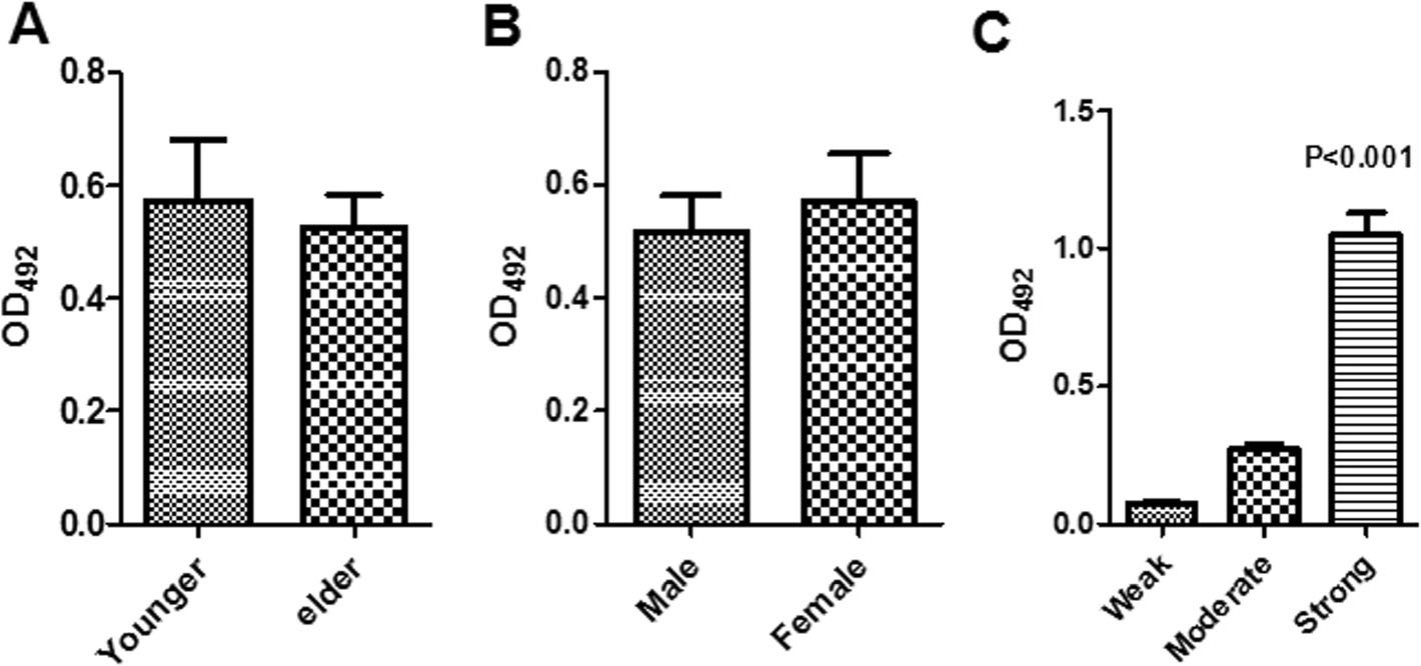
Biofilm formation abilities of *S. maltophilia* in the different genders and ages. Histogram illustrating the ability of biofilm formation. There are no differences in different genders and ages. The number of isolates that formed strong biofilms, however, was significantly greater than the weak and moderate ones

The average biofilm formation ability of *S. maltophilia* was OD_492_ = 0.54 ± 0.49 (0.044–2.34). The OD values of *S. maltophilia* isolated from the male and female patients were OD_492_ of 0.52 ± 0.51 and OD_492_ of 0.57 ± 0.47, respectively, and we did not find any significant difference between the two groups. We also did not find a significant difference in the biofilm formation ability between people age 60 or older and those younger than 60 years old, as shown in Fig. 3. In addition, we analyzed the drug resistance and biofilm formation ability of the isolates and did not find an obvious correlation between the drug-resistant phenotype and the biofilm formation ability, as shown in.

**Fig. 4:**
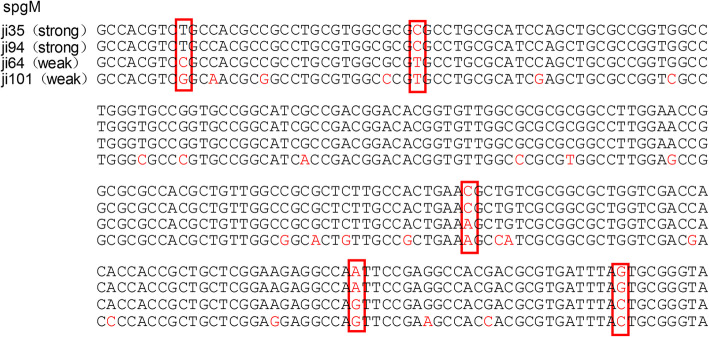
The point mutation of *spgM* in bacterial isolates with different biofilm formation abilities. The *spgM* gene mutations in isolates with strong biofilm formation abilities are significantly different from that with the weak ones. The mutated portions of the DNA bases are shown in the red box

Table 2. The positive rates of the three biofilm genes *rmlA*, *spgM*, and *rpfF* were 82.8% (77/93), 92.5% (86/93), and 64.5% (60/93), respectively. The point mutations of the *spgM* gene in the isolates with strong biofilm formation abilities were relatively consistent and significantly different from those with weak biofilm formation abilities. The detailed sequencing results of some of the isolates are shown in Fig. 4 (the base pairs of the two isolates with different biofilm formation abilities were selected as the representatives). The other two biofilm genes, however, did not have obvious point mutations in the isolates with different biofilm formation abilities.

**Table 2:** Drug-resistant rates and the relationship between the drug resistance and biofilm formation

Antibiotics	Resistant rate	Pearson’s correlation
Levofloxacin	4.3%	0.02
TMP/SMX	9.7%	0.04
Minocycline	0%	NA

### The carriage of the virulence genes

The carriage of the four virulence genes *Stmpr1*, *Stmpr2*, *smf-1*, and *Smlt3773locus* were 79.6, 91.4, 94.6, and 52.7%, respectively.

### Analysis of the risk factors in ICU patients infected with *S. maltophilia*

The results of a univariate analysis demonstrated that the changes in lymphocytes, albumin, and the use of antibiotics were infection risk factors in the ICU patients (Table 3). After the multivariate analysis, we found the type of antibiotic use and lymphocyte count to be independent risk factors of infection with *S. maltophilia* (Table 4). These findings may be used as a new reference index for clinical sensitivity and control of *S. maltophilia*.

**Table 3:** Univariate analysis of risk factors of *S. maltophilia* infections in the ICU

Items	Patients (***n*** = 30)	Control (***n*** = 60)	***P*** value	OR(95%CI)
Male (sex)	23 (76.7%)	38 (63.3%)	0.263	0.565 (0.208–1.534)
Age (years)	64.8 ± 19.1	65.5 ± 16.9	0.873	
Leukocyte	11.5 ± 5.4	10.9 ± 4.1	0.777	
Neutrophil	9.4 ± 4.9	9.4 ± 3.9	0.767	
Lymphocyte	1.3 ± 0.9	0.9 ± 0.4	0.012	
Monocyte	0.7 ± 0.5	0.5 ± 0.4	0.536	
Albumin	30.6 ± 4.2	28.3 ± 5.7	0.033	
Globulin	29.0 ± 6.4	28.2 ± 7.0	0.286	
Prealbumin	129.0 ± 52.3	124.8 ± 49.9	1.000	
Surgeries	14 (46.7%)	27 (45.0%)	0.496	0.724 (0.286–1.835)
Organ transplantation	5 (16.7%)	9 (15.0%)	0.987	0.990 (0.296–3.310)
Malignant tumor	8 (26.7%)	15 (25.0%)	0.894	0.933 (0.337–2.585)
Hypertension	7 (23.3%)	15 (25.0%)	0.923	0.949 (0.328–2.748)
Diabetes	3 (10.0%)	9 (15.0%)	0.397	0.547 (0.135–2.213)
Pulmonary infection	9 (30.0%)	16 (26.7%)	0.990	1.007 (0.374–2.712)
Cardiopathy	4 (13.3%)	9 (15.0%)	0.841	0.875 (0.238–3.213)
Liver injury	4 (13.3%)	7 (11.7%)	0.972	1.024 (0.272–3.856)
Trachea intubation	12 (40.0%)	21 (35.0%)	0.941	1.036 (0.406–2.640)
Chemotherapy	2 (6.7%)	3 (5.0%)	0.843	1.205 (0.189–7.681)
Immunosuppressor	9 (30.0%)	17 (28.3%)	0.867	0.920 (0.343–2.464)
Number of antibiotics	3.6 ± 1.2	3.0 ± 1.1	0.029	
Carbapenems	21 (70.0%)	40 (66.7%)	0.731	1.187 (0.445–3.167)
Cephalosporins	20 (66.7%)	45 (75.0%)	0.604	0.771 (0.289–2.059)
Quinolones	16 (53.3%)	30 (50.0%)	1.000	1.000 (0.396–2.523)

**Table 4:** Multivariate logistic regression analysis associated with *S. maltophilia* infections in the ICU

Risk factors	B value	Wals	***P*** value	OR value	95% CI	
					lower limit	upper limit
Lymphocyte	1.077	4.208	0.04	2.937	1.049	8.222
Albumin	0.099	3.05	0.081	1.104	0.988	1.234
Antibiotics	0.596	5.956	0.015	1.814	1.124	2.927

## Discussion

*S. maltophilia* is an environmental globally emerging gram-negative multidrug-resistant organism that most commonly is associated with respiratory infections in human beings [2, 10]. It is also responsible for many other infectious diseases, including bacteremia, endocarditis, and urethral infection, especially among the immunocompromised, as well as those undergoing aggressive treatments [1]. In patients with pneumonia infections, the mortality rate can range from 14% to as high as 69% [11]. In recent years, *S. maltophilia* has been ranked third among nonfermentative gram-negative bacteria according to the CHINET monitoring service and has been relatively stable, following *Pseudomonas aeruginosa* and *Acinetobacter baumannii*, but its isolation rate has displayed an increasing trend [5]. According to the results of this study, patients age 60 and older were more susceptible to *S. maltophilia* infection, which may have been related to their immunosuppression. Most of the patients had underlying diseases, and 37.6% of the patients had previously invasive examinations or treatments. In addition, there were relatively more male patients. These findings should remind clinicians to pay more attention to the prevention of *S. maltophilia* infections for certain population groups.

The sequence types of the 93 isolates were quite scattered, which indicated that these isolates had loose associations, and thus outbreaks of *S. maltophilia* infection typically have not occurred. By using the MLST results, we easily conducted a comparison with results from other researchers. Note, however, that the PFGE was more about detecting outbreaks in the same hospital. The primary STs in this study were not consistent with those reported in other countries, indicating that isolates from our hospital were quite different from those in other countries. In addition, the consistency between the MLST and PFGE results was poor, and the same ST genotype had no similarity with the PFGE typing. This result indicated that the *S. maltophilia* isolates had genetic diversity, which was consistent with the results of foreign studies regarding the differences between the two detection methods [12–14].

All of the patients had antibiotic usage (one to six types) before the isolation, of which, 69 patients had used antibiotics three or more times. The majority of antibiotics included cephalosporins, carbapenem, and β-lactamase inhibitors. The use, however, of cephalosporins and carbapenem can easily cause *S. maltophilia* to be selected as the dominant flora. According to the epidemic characteristics of *S. maltophilia*, it is believed that *S. maltophilia* infection is an endogenous infection under the interaction between drug selection and its own environment, rather than an interpersonal infection in a ward [2].

*S. maltophilia* exhibits complicated resistance to a broad array of antibiotics, limiting available therapeutic options. Because *S. maltophilia* is intrinsically resistant to a variety of antibiotics, we did not perform a drug resistance analysis to other drugs in this study. We did analyze, however, three commonly used antimicrobial agents against *S. maltophilia* infection in clinic. The results of the antibiotic susceptibility test showed that the *S. maltophilia* isolates in this study were susceptible to three targeted antimicrobial agents in the clinic, and the drug resistance rate was low. One isolate, however, was still resistant to two of the tested antibiotics. Because of the existence of complicated multidrug-resistant mechanisms in *S. maltophilia* and the worsening coinfection phenomenon, diligence to this infection is still needed, especially in monitoring the multidrug-resistance of *S. maltophilia*. In the analysis of risk factors, in addition to the analysis of common factors, we specifically examined indicators related to immunosuppression, such as leukocytes, neutrophils, and immunoglobulins. Leukocytes and neutrophils are greatly affected by outside influences, however, and the number will rise following a bacterial infection. We speculated that lymphocytes (which are less affected by stress and microbial factors), immunoglobulin, albumin, and prealbumin affected by nutritional factors, may be used as observable indicators. The results also showed that lymphocytes and albumin were independent risk factors of *S. maltophilia* infection, and lymphocytes could be used as an independent factor to provide an important method and basis to clinically guide the monitoring, prevention, and control of *S. maltophilia*. In the *S. maltophilia* infection group, the concentration of albumin was higher than that in the control group. Whether this was caused by compensation or by other factors, however, still needs to be examined. In this study, we did not accurately record the number of times that carbapenem was used or that endotracheal intubation occurred, which likely were risk factors. This was a major defect of this study, and a supplemental study to investigate these two indicators will be performed in the future. This study also had some other inadequacies. We found that mutations in the *spgM* gene in the isolates with strong biofilm formation abilities were significantly different from those with weak biofilm formation abilities. We did not, however, investigate the exact mechanisms. This aspect requires further attention in a future study.

## Conclusions

The genotyping of the isolates showed high diversity, indicating the distant correlation of these isolates and the low-occurring clonal transmission of *S. maltophilia* in the same department. This result suggested that clinical *S. maltophilia* infections are perhaps endogenous infections under antibiotic selection. This conclusion is a reminder that antibiotics should be reasonably used to reduce the incidence of infection. We found that the types of antibiotic use and lymphocyte counts were independent risk factors of *S. maltophilia* infection. The mutations in the *spgM* gene were associated with biofilm-forming abilities, which is worthy of future research. Because of the strong biofilm formation ability and high virulence gene carrying rate, high-risk patient groups should receive more attention to avoid potential risk of *S. maltophilia* infection.

## Methods

### Materials and reagents

We collected a total of 93 nonrepetitive isolates of *S. maltophilia* from outpatients and inpatients from Renji Hospital in 2014, in addition to the patients’ clinical information. We identified these isolates using the VITEK-2 Compact System (bioMérieux, Marcy-l’Étoile, France) and confirmed them using matrix-assisted laser desorption ionization time-of-flight mass spectrometry (MALDI-TOF MS, Bruker Daltonics, Bremen, Germany). The samples were then stored at minus 80 °C.

We grew the bacteria in tryptic soy broth (TSB; Oxoid, Hampshire, UK) at 37 °C overnight. The antibiotics used for disk diffusion testing were levofloxacin, TMP/SMX, and minocycline (Oxoid). The quality control isolates included *Escherichia coli* ATCC25922 and *Pseudomonas aeruginosa* ATCC27853.

### Disk diffusion testing

We performed disk diffusion according to the Clinical and Laboratory Standards Institute (CLSI) 2019 recommendations for levofloxacin, TMP/SMX, and minocycline.

### Multilocus sequence typing analysis

We used primer sequences targeting the conserved regions of seven housekeeping genes of *S. maltophilia*, as shown on the multilocus sequence typing (MLST) site (http://pubmlst.org/smaltophilia/). We performed MLST as described by Kaiser et al. [15].

Seven pairs of primers for the housekeeping genes (*atpD*, *gapA*, *guaA*, *mutM*, *nuoD*, *ppsA,* and *recA*) were synthesized (Supplementary Table 1). We incubated the polymerase chain reaction (PCR) mixture (2×PCR mix, primers, DNA template, and double distilled water) at 94 °C for 5 min; 35 cycles at 94 °C for 10 s, 55 °C for 30 s, and 72 °C for 30 s, with a final extension step at 72 °C for 1 min. We analyzed the sequence using DNAstar software and submitted the obtained sequence to the MLST database to acquire the sequence type. We assembled the seven housekeeper genes using MEGA.4 software and conducted a phylogenetic analysis.

### Pulsed-field gel electrophoresis analysis

We performed the pulsed-field gel electrophoresis (PFGE) analysis using the Bio-Rad system and slightly modified the protocol according to the Tanimoto’s and Shueh’s reports [14, 16]. We conducted the preliminary experiments as follows: Restriction enzyme Xbal (Roche Diagnostics, Basel, Switzerland) was digested at 37 °C for 3 h. The PGFE electrophoresis conditions followed the manufacturer’s protocol: 2000 mL 0.5 *TBE, voltage of 6.0 V max, temperature of 14 °C, pulse angle of 120, start pulse time of 5 s, end pulse time 20 s, and an electrophoresis time of 19 h. We analyzed the results using BioNumerics software (version 4.0, Applied Maths, Sint-Martens-Latem, Belgium). The *Salmonella* serotype Braenderup strain (H9812) was the molecular weight marker.

### Biofilm formation assay

We diluted the overnight cultured *S. maltophilia* was diluted in TSB to D_600_ of 0.01. A total of 200 μl of the solution in each well was cultured at 37 °C for 24 h in a 96-well plate (Corning, Corning, NY, USA). We determined biofilm formation ability using dye crystal violet staining. After incubation, the plate was fixed at 60 °C for 1 h. The nonadherent bacteria was removed and washed with sterile phosphate buffer solution (PBS) four times, then 50 μL of crystal violet dye was added to each well and kept at room temperature for 5 min. This was followed by rinsing under running tap water. The plate was dried at room temperature and 250 μL of 33% glacial acetic acid was added to each well to dissolve the staining for 15 min. To measure the absorbance, the optical density was read at 492 nm. We defined a low cut-off (ODc) as 3× standard deviation (SD) above the mean OD of the control wells. We classified the biofilm formation ability as follows: no biofilm production (OD ≤ ODc), weak biofilm production (ODc < OD ≤ 2 × ODc), moderate biofilm production (2 × ODc < OD ≤ 4 × ODc), and strong biofilm production (4 × ODc < OD).

### Detection of biofilm and virulence genes

We amplified three biofilm genes, *rmlA*, *spgM*, and *rpfF*, and four virulence genes, *Stmpr1*, *Stmpr2*, *smf-1*, and *Smlt3773*, using PCR. We performed PCR as previously described in the MLST section, and these genes’ primers are shown in Supplementary Table 1.

### Risk factor analysis

We collected the clinical information of 30 patients with *S. maltophilia* infection from the ICU. Each *S. maltophilia*-infected patient was matched with two patients without *S. maltophilia* infection from the same department during the same period and with age differences of less than 3 years. In addition, we analyzed the blood infection routines and the results of their biochemical tests. Additionally, we combined clinical diseases, operations, treatments, and other items for each patient to analyze the risk factors of *S. maltophilia* infection in patients from the ICU.

### Statistical analysis

We used SPSS 22 (IBM SPSS Statistics for Windows 22.0, IBM Corp. Armonk, NY, USA) and GraphPad Prism 8 (GraphPad software Inc.; San Diego, CA, USA) for the data processing. We analyzed the normality using the Shapiro-Wilk test. We used the median and range (or mean ± SD) and a one-way analysis of variance (ANOVA) for the continuous variables and used a chi-square test for the categorical data. We used percentages (%)for the positive rates, such as the drug resistance rate, biofilm gene positive rate, and virulence gene positive rate. We considered values of *p* < 0.05 to be statistically significant. We compared the correlation between the clonal typing and drug resistance rate and compared the correlation between the biofilm and drug resistance rate using the Pearson correlation coefficients.

## Supplementary information

**Additional file 1: Figure S1.** Dendrogram of the obtained PFGE XbaI profiles of *S. maltophilia* clinical isolates. The distance shown above the dendrogram represents the genetic relatedness between the analyzed isolates.

**Additional file 2: Table S1.** Primers for MLST, biofilm, and virulence genes of *S. maltophilia.*

## Data Availability

The data used and analyzed during the current study are available from the first author on reasonable request.

## Abbreviations

*S. maltophilia*: *Stenotrophomonas maltophilia*
PCR: Polymerase chain reaction
ATCC: American Type Culture Collection
MIC: Minimum inhibitory concentration
MLST: Multilocus sequence typing
PFGE: Pulsed field gel electrophoresis
ST: Sequence type
TMP/SMX: Trimethoprim/sulfamethoxazole
TSB: Tryptic soy broth
OD: Optical density
CLSI: Clinical and Laboratory Standards Institute
PBS: Phosphate buffer solution
