# Supplementary figures and images for: Molecular epidemiology and risk factors of Stenotrophomonas maltophilia infections in a Chinese teaching hospital
Source: BMC Microbiol. 2020 Sep 29;20:294. doi: 10.1186/s12866-020-01985-3 (PMC7526397; doi:10.1186/s12866-020-01985-3)

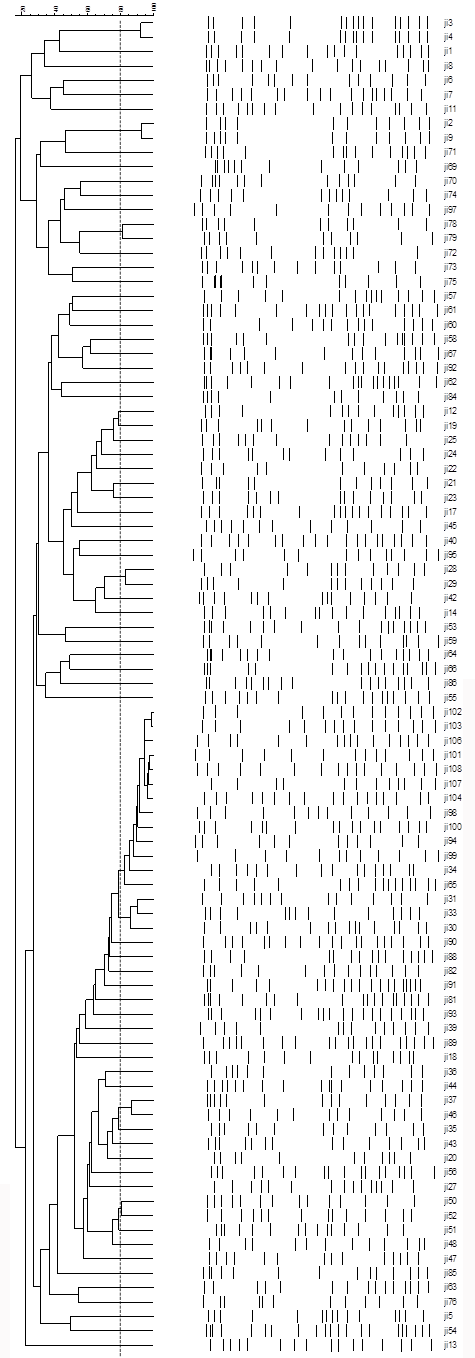

Supplement: Supplementary file 1 — Additional file 1: Figure S1. Dendrogram of the obtained PFGE XbaI profiles of S. maltophilia clinical isolates. The distance shown above the dendrogram represents the genetic relatedness between the analyzed isolates. [file 12866_2020_1985_MOESM1_ESM.tif]
