# Supplementary material for: Molecular epidemiology and risk factors of Stenotrophomonas maltophilia infections in a Chinese teaching hospital
Source: BMC Microbiol. 2020 Sep 29;20:294. doi: 10.1186/s12866-020-01985-3 (PMC7526397; doi:10.1186/s12866-020-01985-3)
Supplement: Supplementary file 2 — Additional file 2: Table S1. Primers for MLST, biofilm, and virulence genes of S. maltophilia. [file 12866_2020_1985_MOESM2_ESM.doc]

**Supplementary Table 1 Primers for MLST and detection of biofilm and virulence genes**

| **Primer** | **Gene** | **Sequence(5'-3')** | **Size(bp)** |
| --- | --- | --- | --- |
| atpD-F | *atpD* | ATGAGTCAGGGCAAGATCGTTC | 858 |
| atpD-R |  | TCCTGCAGGACGCCCATTTC |  |
| gapA-F | *gapA* | TGGCAATCAAGGTTGGTATCAAC | 800 |
| gapA-R |  | TTCGCTCTGTGCCTTCACTTC |  |
| guaA-F | *guaA* | AACGAAGAAAAGCGCTGGTA | 704 |
| guaA-R |  | ACGGATGGCGGTAGACCAT |  |
| mutM-F | *mutM* | AACTGCCCGAAGTCGAAAC | 579 |
| mutM-R |  | GAGGATCTCCTTCACCGCATC |  |
| nuoD-F | *nuoD* | TTCGCAACTACACCATGAAC | 514 |
| nuoD-R |  | CAGCGCGACTCCTTGTACTT |  |
| ppsA-F | *ppsA* | CAAGGCGATCCGCATGGTGTATTC | 635 |
| ppsA-R |  | CCTTCGTAGATGAA(A/G)CCGGT(A/G)TC |  |
| recA-F | *recA* | ATGGACGAGAACAAGAAGCGC | 807 |
| recA-R |  | GGTGATGACCTGCTTGAACGG |  |
| rmlA-F | *rmlA* | CGGAAAAGCAGAACATCG | 1222 |
| rmlA-R |  | GCAACTTGGTTTCAATCACTT |  |
| spgM-F | *spgM* | ATACCGGGGTGCGTTGAC | 2750 |
| spgM-R |  | CATCTGCATGTGGATCTCGT |  |
| rpfF-F | *rpfF* | CACGACAGTACAGGGGACC | 1140 |
| rpfF-R |  | GGCAGGAATGCGTTGG |  |
| Stmpr1-F | *Stmpr1* | TGGATGCTTGGTCCCGTAGT | 2540 |
| Stmpr1-R |  | CCGTGGTGTCGGCTTCGATCTCT |  |
| Stmpr2-F | *Stmpr2* | GCCGATTCCGGCATTCACACC | 1764 |
| Stmpr2-R |  | GGTCAGGCCCGAGAAGGTGCT |  |
| Smf-1-F | *Smf-1* | GGAAGGTATGTCCGAGTCCG | 674 |
| Smf-1-R |  | GCGGGTACGGCTACGATCAGTT |  |
| Smlt3773 | *Smlt3773* | CGGTGCCGAACTCGTAACCGG | 1342 |
| locus-F | *locus* |  |  |
| Smlt3773 |  | CTTCCGGCCATGGCAGGCGAA |  |
| locus-R |  |  |  |
